# Examining a DNA Replication Requirement for Bacteriophage λ Red- and Rac Prophage RecET-Promoted Recombination in *Escherichia coli*

**DOI:** 10.1128/mBio.01443-16

**Published:** 2016-09-13

**Authors:** Lynn C. Thomason, Nina Costantino, Donald L. Court

**Affiliations:** a Basic Science Program, GRCBL-Molecular Control and Genetics Section, Frederick National Laboratory for Cancer Research, Leidos Biomedical Research, Inc., Frederick, Maryland, USA; b Gene Regulation and Chromosome Biology Laboratory, National Cancer Institute, Frederick, Maryland, USA

## Abstract

Recombineering, *in vivo* genetic engineering with bacteriophage homologous recombination systems, is a powerful technique for making genetic modifications in bacteria. Two systems widely used in *Escherichia coli* are the Red system from phage λ and RecET from the defective Rac prophage. We investigated the *in vivo* dependence of recombineering on DNA replication of the recombining substrate using plasmid targets. For λ Red recombination, when DNA replication of a circular target plasmid is prevented, recombination with single-stranded DNA oligonucleotides is greatly reduced compared to that under replicating conditions. For RecET recombination, when DNA replication of the targeted plasmid is prevented, the recombination frequency is also reduced, to a level identical to that seen for the Red system in the absence of replication. The very low level of oligonucleotide recombination observed in the absence of any phage recombination functions is the same in the presence or absence of DNA replication. In contrast, both the Red and RecET systems recombine a nonreplicating linear dimer plasmid with high efficiency to yield a circular monomer. Therefore, the DNA replication requirement is substrate dependent. Our data are consistent with recombination by both the Red and RecET systems occurring predominately by single-strand annealing rather than by strand invasion.

## IMPORTANCE

Bacteriophage homologous recombination systems are widely used for *in vivo* genetic engineering in bacteria. Single- or double-stranded linear DNA substrates containing short flanking homologies to chromosome targets are used to generate precise and accurate genetic modifications when introduced into bacteria expressing phage recombinases. Understanding the molecular mechanism of these recombination systems will facilitate improvements in the technology. Here, two phage-specific systems are shown to require exposure of complementary single-strand homologous targets for efficient recombination; these single-strand regions may be created during DNA replication or by single-strand exonuclease digestion of linear duplex DNA. Previously, *in vitro* studies reported that these recombinases promote the single-strand annealing of two complementary DNAs and also strand invasion of a single DNA strand into duplex DNA to create a three-stranded region. Here, *in vivo* experiments show that recombinase-mediated annealing of complementary single-stranded DNA is the predominant recombination pathway in *E. coli*.

## INTRODUCTION

**FIG 1:**
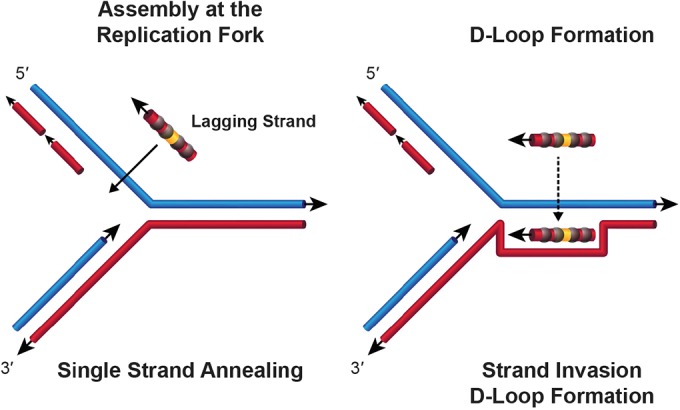
Models of oligonucleotide recombination. (A) Single-strand annealing model of oligonucleotide recombination at DNA replication fork. The oligonucleotide is bound by the recombinase and annealed to complementary ssDNA arising at the replication fork. Larger gaps on the lagging strand because of discontinuous synthesis promote recombination with the lagging-strand oligonucleotide and give rise to the lag-lead bias. (B) Model of strand invasion. The oligonucleotide is bound by the recombinase, which locates homologous dsDNA and opens the DNA duplex, annealing the oligonucleotide to its complement and displacing the DNA that is identical in sequence, forming a D-loop.

The bacteriophage λ Red and the bacterial Rac prophage RecET generalized recombination systems are used for efficient insertion of short linear DNAs, a process known by its colloquial name, recombineering (1–5). Each system has a highly processive 5′-to-3′ double-stranded DNA (dsDNA) exonuclease, λ Exo (6, 7) or Rac RecE (8–10), respectively, and single-strand annealing proteins (recombinase), λ Beta (11, 12) or Rac RecT (13, 14), respectively. *In vitro*, both recombinases have been reported to promote single-strand annealing, as well as strand invasion, the latter only at very AT-rich sequences (Fig. 1A and B) (15, 16).

When used for recombineering, Beta, RecT, and other phage recombinases give robust levels of recombination with short single-stranded DNA (ssDNA) oligonucleotides (3, 17). Additionally, a basal level of this type of homologous ssDNA recombination occurs in *Escherichia coli* and other bacterial species in the absence of any known host or phage recombination system (18–20). For dsDNA recombination, the exonuclease and recombinase pairs act in concert to process and recombine linear dsDNA; in this reaction, exonuclease digestion results in 3′ single-strand overhangs that are recombined by the recombinases (1, 2). Linear dsDNA recombination is enhanced by the λ Gam protein, which inhibits the *E. coli* RecBCD exonuclease (17, 21, 22).

Classical experiments with phage λ infections (23–25) found that Red and RecET behaved similarly when promoting phage λ recombination, with both showing a dependence on DNA replication, and it was suggested that phage recombination events may occur best at replication forks or on newly replicated DNA (25). When λ DNA replication was prevented, Red recombination was limited and occurred only near double-strand breaks in the phage DNA (26). This recombination was subsequently shown to occur predominately by single-strand annealing (27). *In vivo*, RecET functions can promote intramolecular circularization of nonreplicating linear DNA molecules containing homologous repeats to generate recombinant plasmids (28–31).

Reports from several labs have suggested that DNA replication of the target molecule is important for recombineering. The first hint came from the observation that Red-mediated ssDNA recombination displays a strand preference, in that one of two complementary strands yields a higher recombinant frequency than the other (3, 32). This strand bias is not dependent upon transcription but is dependent upon the direction of DNA replication through the target, such that the oligonucleotide conferring the higher recombination frequency corresponds in sequence to the discontinuously replicated lagging strand. A lagging/leading bias was also observed with RecT (17), as well as with other, similar, recombinase functions. Efficient recombineering with the Red system requires functions involved in DNA replication (33–36). If an intact plasmid target cannot replicate, the level of Red-mediated ssDNA and dsDNA recombination is reduced (33, 36, 37).

We have further investigated the involvement of DNA replication of the target plasmid for oligonucleotide recombination, both that mediated by the λ Red or Rac RecET recombinase and that which occurs in the absence of any known recombinase. First, we assayed single-strand oligonucleotide recombination targeting pUC plasmids that were either freely replicating or blocked for DNA replication. Second, we examined Red- and RecET-dependent circularization of a nonreplicating linear plasmid containing a direct repeat. Our experimental results are consistent with the Red Beta and Rac RecT recombinases acting predominately by single-strand annealing rather than by strand invasion, whether they are targeting circular plasmids or linear substrates.

## RESULTS AND DISCUSSION

### Oligonucleotide-plasmid recombination.

For supercoiled plasmids, the phage recombinase, i.e., Red Beta or Rac RecT, must initiate recombination with ssDNA on the circular substrate (Fig. 1). If these proteins act solely by single-strand annealing, they will require ssDNA targets on the plasmid for this initiation. Such targets arise as the replication fork passes, generating a long ssDNA region on the lagging-strand template and a much shorter ssDNA region ahead of the continuously replicated leading strand (3). In the absence of plasmid replication, these targets are absent. In contrast, initiation of recombination by strand invasion does not require a single-stranded target and should be independent of DNA replication. If strand invasion occurs, duplex DNA is opened; the oligonucleotide is annealed by the recombinase to the complementary target, and the displaced DNA is extruded in a D-loop. The D-loop can be further processed to form complete recombinants. Events initiated by strand invasion should happen at the same frequency whether or not DNA replication occurs and with equal probability on either strand.

### Experimental design.

To address these models, we compared ssDNA oligonucleotide recombination frequencies targeting either freely replicating or nonreplicating plasmids. We used bacterial hosts expressing either Red Beta or Rac RecT or lacking these recombinases. We used a *polA resA1* mutation (38) to block origin-dependent plasmid replication (39). This mutation does not prevent replication of the *E. coli* chromosome or prevent Red-mediated recombination for oligonucleotide correction of a point mutation on the *E. coli* chromosome (34). The strains are RecA^+^; however, the oligonucleotide recombination that occurs in these strains is independent of RecA (3, 18, 19, 40). Detecting recombinants in replication-blocked experiments requires isolation of plasmid DNA after the cells are washed to remove any extracellular nucleic acids. Isolated plasmid DNA is then introduced into replication-competent cells lacking any recombination function, where previously initiated recombinants can be scored. When plasmid replication is allowed, in the PolA^+^ host, recombination frequencies can be scored either by plating cells directly on selective medium or by the same DNA isolation and transformation procedure used for the *polA resA1* mutant strains.

### Plasmid substrates for ssDNA oligonucleotide recombination.

We used two plasmid substrates with useful properties for our experiments. Plasmid pLT60 is a pUC derivative carrying *bla* conferring Amp^r^ and the *kan* gene carrying an amber mutation at tyrosine codon 39 [*kan*(Am)] (41). Plasmid pLT62 is identical to pLT60, except that it contains a four-base replacement (CGAG) of a three-base sequence (AGC) in *kan* at the 39th codon, resulting in a frameshift and introducing a unique XhoI restriction site. These multiple mispairs are refractory to mismatch repair (MMR) (32, 40), and equivalent numbers of recombinants are obtained whether recombination is executed in strains with or without MMR functions. In our assays, the plasmid mutations are repaired to *kan*^+^ conferring kanamycin resistance (Kan^r^). A set of two complementary ssDNA oligonucleotides, LT217 and LT213 (see Table S1 in the supplemental material), were used to repair the mutations. LT217 is a lagging-strand oligonucleotide where the lagging strand is the same as the discontinuously replicated strand. LT213 is a leading-strand oligonucleotide where the leading strand is the same as the continuously replicated strand. In all cases, plasmid DNA and oligonucleotides were introduced by coelectroporation (see Materials and Methods for details).

### Repairing a point mutation on a plasmid in PolA^+^ cells.

**FIG 2:**
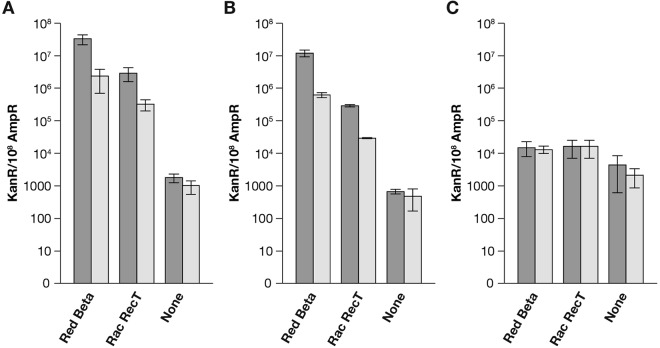
Repair of a point mutation with an ssDNA oligonucleotide on a freely replicating plasmid. pLT60 was used for plasmid-oligonucleotide crosses in strains HME68 (Red Beta), SIMD89 (Rac RecT), and LT1714 (no recombinase). (A) The dark bars show recombination frequencies as numbers of Kan^r^ plasmids per 10^8^ Amp^r^ transformants scored by direct plating with the lagging-strand oligonucleotide, LT217. The lighter bars show recombinant frequencies for the leading-strand oligonucleotide, LT213. (B) The dark and lighter bars show recombination frequencies for the same replicating crosses, but in this case, plasmid DNA was isolated and then introduced into DH10B. Here, total plasmid transformants and recombinants were scored and the frequency of recombinants is shown. (C) Repair of a point mutation with an ssDNA oligonucleotide on a nonreplicating plasmid. pLT60 was used for plasmid-oligonucleotide crosses executed in *polA* mutant hosts HME69 (Red Beta), LT1712 (Rac RecT), and LT1715 (recombinase independent). The dark and lighter bars show recombination frequencies obtained following plasmid DNA isolation and transformation in DH10B *mutS* for the lagging-strand oligonucleotide LT217 and the leading-strand oligonucleotide LT213, respectively. In all cases, error bars indicate the standard error of the mean.

Recombination frequencies for freely replicating plasmid pLT60 by oligonucleotide crosses were determined by plating cells on selective media in hosts expressing the Red Beta or Rac RecT recombinase or neither phage recombinase (Fig. 2A; see Table S2 in the supplemental material), scoring the frequency of Kan^r^ among total Amp^r^ plasmid transformants. For the recombinase-expressing strains, the recombination frequencies we observed here with plasmid pLT60 are consistent with our previous results obtained with lagging and leading oligonucleotides to target *galK* on the *E. coli* chromosome (3, 17, 32, 40). For Red Beta, >30% of the total colonies contained lagging-strand recombinant plasmids, about 10-fold more than we observed for RecT. Without any recombinase, the level of lagging-strand recombinants was >10,000-fold lower than that obtained with Red. For the recombinases, the observed lag/lead bias was similar to that observed for recombineering when targeting the bacterial chromosome (19). No significant lag/lead bias was seen when the recombinase-independent strain was used.

### Isolation and reintroduction of plasmid DNA from replicating crosses.

A plasmid DNA isolation procedure is necessary in order to score oligonucleotide recombination that initiated in the absence of plasmid replication in the *polA* mutant hosts. We also isolated freely replicating plasmid DNA from the PolA^+^ hosts and introduced that DNA into a second bacterium lacking recombination functions at less than one molecule per cell. We found a lower apparent recombination frequency when the plasmids were assayed in the second host than with direct plating as described above. This is because many cells from which plasmids were isolated contained no recombinants, only parental plasmids. Even cells with recombinants contained a mixture of parental and recombinant species, with recombinants in the minority, as measured previously (41). The apparent reductions in recombination frequencies with this procedure were ~3-fold for Red Beta, ~10-fold for RecT, and 2- to 3-fold when the cells lacked a phage recombinase (compare Fig. 2A and B; see Table S2 in the supplemental material). We will compare data from these experiments in which replication was allowed to those from the experiments in which *polA* replication was blocked.

### Repairing a point mutation on a plasmid in *polA resA1* mutant cells.

For both phage recombinases, when plasmid DNA replication was allowed, the lagging-strand oligonucleotide, LT217, gave at least 10-fold more recombinants than the leading-strand oligonucleotide, LT213. In contrast, the basal recombinase-independent level of plasmid recombination displayed no significant strand bias. When DNA replication is prevented for Beta, RecT, or the recombinase-independent systems, the lagging-versus-leading-strand bias is abolished, with the two complementary oligonucleotides giving nearly identical frequencies (Fig. 2C; see Table S3 in the supplemental material). In the absence of plasmid DNA replication, expression of Beta or RecT increased recombination only marginally above that observed in the absence of the recombinases, with that level converging to ~1 × 10^4^/10^8^ total plasmid transformants (Fig. 2C; see Table S3). Thus, frequencies of Beta and RecT recombination were both reduced by blocking replication but Beta recombination was more strongly impacted (compare Fig. 2B and C). For Beta, the reduction was more than 800-fold for the lagging-strand oligonucleotide. In contrast, for RecT, the efficiency of lagging-strand oligonucleotide recombination was reduced <20-fold and leading-strand recombination had little or no dependence on replication. The recombination that occurred in the absence of the phage recombinases was ~5-fold higher when plasmid replication was blocked (compare Fig. 2B and C). If recombinase-independent formation of recombinant plasmids is a slow process, molecules that are undergoing recombination could be diluted out by freely replicating plasmids.

### Multiple adjacent mismatches reduce recombination levels.

**FIG 3:**
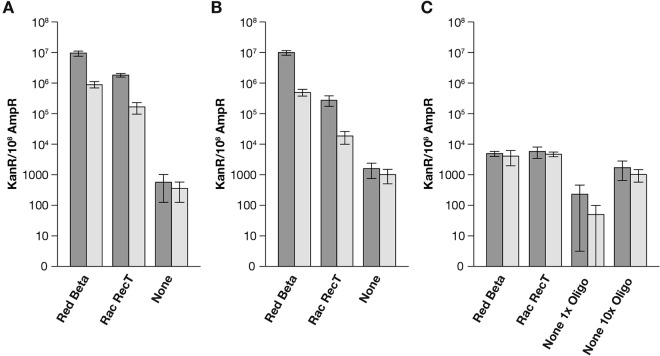
Repair of multiple contiguous mispairs with an ssDNA oligonucleotide on a freely replicating plasmid. Plasmid pLT62, containing four mismatches, was used for plasmid-oligonucleotide crosses in strains HME68 (Red Beta), SIMD89 (Rac RecT), and LT1714 (recombinase independent). (A) The dark bars show recombination frequencies as numbers of Kan^r^ plasmids per 10^8^ Amp^r^ transformants scored by direct plating with the lagging-strand oligonucleotide LT217. The lighter bars show recombinant frequencies for the leading-strand oligonucleotide LT213. (B) The dark and lighter bars show recombination frequencies for the same replicating crosses, but in this case, plasmid DNA was isolated and then introduced into DH10B. Here, the total numbers of plasmid transformants and recombinants were scored and the frequency of recombinants is shown. (C) Repair of multiple contiguous mispairs with an ssDNA oligonucleotide on a nonreplicating plasmid. pLT62-ssDNA oligonucleotide crosses were executed in *polA* mutant hosts HME69 (Red Beta), LT1712 (Rac RecT), and LT1715 (recombinase independent). The dark and lighter bars show recombination frequencies scored in DH10B for the lagging-strand oligonucleotide LT217 and the leading-strand oligonucleotide LT213, respectively. For LT1715, the first data set was obtained with 5 pmol of oligonucleotide while the second data set was obtained with 50 pmol of oligonucleotide. Error bars indicate the standard error of the mean.

It was previously demonstrated (34, 40) that multiple mispaired bases in the oligonucleotides are repaired by the Red system, but with reduced recombination efficiency. Experiments similar to those described for pLT60 were done with plasmid pLT62 (Fig. 3A to C; see Tables S4 and S5 in the supplemental material), which contains four contiguous base changes, with a frameshift at the site of the amber mutation in pLT60. The oligonucleotides, when annealed to pLT62, form a larger mismatch, with four bases on the plasmid and three on the oligonucleotide remaining unpaired. When assayed directly, the replication-allowed recombinase-mediated recombination frequencies obtained with pLT62 are lower (~4-fold) than those found with pLT60, consistent with previous experiments (34, 40) (compare Fig. 2A and 3A). In the absence of any phage recombinase, a greater reduction (~15-fold) in the repair of multiple mismatches versus a single base mismatch was observed. Thus, basal recombination with a substrate containing multiple mismatches is lower than that that of a substrate with a point mutation, indicating that however the basal level of recombination occurs, the process is inhibited more by several contiguous mispairs than are the recombinase-mediated reactions. This observation is in keeping with the known *in vitro* properties of Beta, which can drive strand exchange through several mismatches (42).

When the point mutation on pLT60 was repaired in the absence of DNA replication (Fig. 2C), the efficiency of recombination observed in the absence of Beta and RecT was only slightly lower than when they were expressed, arguing against the idea that these functions stimulate recombination on a nonreplicating template. The background level of recombination obscures most of the activity the recombinases might have under these conditions. In contrast, when replication of pLT62 was prevented, only a very few Kan^r^ recombinants were recovered from recombinase-independent experiments (Fig. 3C). Our results show that although oligonucleotides containing multiple adjacent mispairs are more difficult to anneal to the complementary target, both Beta and RecT can promote this recombination on a nonreplicating substrate. A 10-fold increase in targeting oligonucleotide allowed isolation of Kan^r^ pLT62 recombinants from the recombinase-deficient *polA* mutant host, with frequencies similar to those seen for the recombinase-independent pLT60 replication-blocked crosses (compare Fig. 2C to 3C). In the absence of a phage recombinase, flooding the cell with excess targeting oligonucleotide improves a poor reaction that may be dependent on mass action. The stimulation observed when the concentration of the targeting oligonucleotide is increased is in contrast to our observations with Beta-dependent recombination targeting pLT62, where adding 10-fold more targeting oligonucleotide did not increase the recombination frequency in the absence of plasmid replication (see Table S5 in the supplemental material).

Both Beta and RecT were initially identified as single-strand annealing proteins lacking the strand invasion activity possessed by RecA protein (12, 13). Later, they were reported to have limited strand invasion activity, but only on AT-rich substrates (15, 43). Since the GC content in the region of our oligonucleotide targets on the plasmid is ~50%, strand invasion by either recombinase is unlikely in our *in vivo* system. When the two recombinases mediate oligonucleotide recombination targeting either of the nonreplicating plasmids, their frequencies converge to the same value. It is theoretically possible that both recombinases promote the same low level of strand invasion on these substrates. However, we think that a more likely interpretation of our data is that a fraction of the intracellular plasmid population has lesions in the DNA. This plasmid DNA was isolated with a commercial kit. Thus, some fraction of the DNA is not supercoiled and likely contains nicks or gaps and may be partially replicated. Lesions that reveal the single-stranded complementary target would allow single-strand oligonucleotides to anneal and initiate recombination, in the presence or absence of recombinases. Once initiation has occurred, the strand exchange properties of the recombinases (42, 44) could promote more efficient incorporation of the oligonucleotide on the nonreplicating plasmid. If this interpretation is correct, we predict that ~10^−4^ of our plasmid DNAs contains ssDNA at the target site. This frequency reflects the number of recombinants (10^4^ Kan^r^) per 10^8^ Amp^r^ transformants.

### Detection of plasmid recombination intermediates formed in the absence of DNA replication.

**FIG 4:**
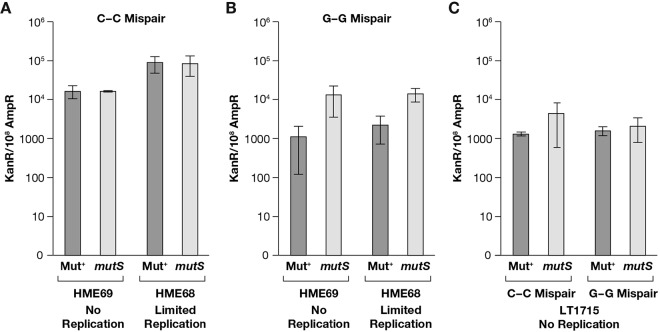
Detection of recombination intermediates genetically. (A) Plasmid DNA was isolated from Red-mediated pLT60 by LT217 crosses performed under two different conditions: replication blocked in host HME69 or replication allowed in host HME68 but with only a single hour of outgrowth. The C-C mispair formed when this oligonucleotide was annealed to its target was refractory to MMR. These DNAs were introduced into either Mut^+^ or *mutS* mutant DH10B derivatives by electrotransformation, where the number of Kan^r^ plasmid recombinants per 10^8^ Amp^r^ transformants was scored. The first set of two columns shows the frequencies obtained in the absence of plasmid replication, while the second set of two columns shows the frequencies obtained when 1 h was allowed for replication. In both cases, the frequencies are the same whether the DH10B host is MMR proficient or deficient. (B) Plasmid DNA was isolated from Red-mediated pLT60 by LT213 crosses performed with the same strains under the same two conditions, and the data are organized as in panel A. The G-G mispair formed when oligonucleotide LT213 is annealed to its target is proficiently repaired by the MMR system. When these DNAs were introduced into MMR-proficient or -deficient DH10B host cells, the frequency of recombinants scored in the Mut^+^ host is reduced 10-fold relative to that in the *mutS* mutant host. These data are consistent with the MMR system repairing a G-G mismatch heteroduplex that remains in the transformed DNA, thus reducing the number of recombinants scored in that host. (C) Plasmid DNA was isolated from recombinase-independent pLT60-LT217 (lagging) and -LT213 (leading) oligonucleotide crosses performed under replication-blocked conditions in host LT1715. This DNA was then introduced into either Mut^+^ or *mutS* mutant DH10B derivatives by electrotransformation, and recombinants were scored as described above. The first two bars show data for the LT217 cross, which results in a C-C mispair, while the last two bars show results for the LT213 cross, which results in a G-G mispair.

Initiation of oligonucleotide recombination in the absence of plasmid DNA replication is expected to form heteroduplex plasmid molecules, with the ssDNA oligonucleotide annealed to the target sequence. These intermediates can be further processed into completed recombinants; one route to their completion is by DNA replication in the second host. We looked for heteroduplex recombination intermediates that formed in the *polA resA1* mutant Beta- and RecT-expressing strains as follows. Plasmid pLT60 DNA preparations, isolated from replication-blocked recombination experiments, were introduced into *recA* mutant strains that were either proficient or deficient in methyl-directed MMR. When oligonucleotide LT217 anneals to its complementary target on pLT60, a C-C mispair is formed; C-C mismatches are not corrected by the MMR system (32, 40, 45), so the apparent recombinant efficiency should be the same whether recombinants are scored in a Mut^+^ or a *mutS* mutant host. This is what we observed (Fig. 4A). In contrast, when oligonucleotide LT213 pairs to its complementary target sequence on pLT60, a G-G mismatch is formed. Since G-G mispairs are well repaired by MMR (32, 45), MMR will occur in the Mut^+^ host to remove G-G heteroduplexes, resulting in a reduction of Kan^+^ colonies relative to those where the *mutS* mutant strain was transformed. For Red Beta, the “apparent” recombination efficiency of oligonucleotide LT213 targeting pLT60 was ~12-fold lower in the Mut^+^ host than that found in the *mutS* mutant host (Fig. 4B; see Table S3 in the supplemental material). This differential efficiency of Beta-mediated LT213 recombinants is indicative of heteroduplex plasmid recombination intermediates that form in the absence of DNA replication and that can be subsequently repaired in the Mut^+^ host when the plasmid is replicated. Methyl-directed MMR of the newly replicated, unmethylated DNA strand is coupled to DNA replication (46). For RecT, we found that the apparent recombination efficiency of oligonucleotide LT213 was ~6-fold lower in a Mut^+^ host than in a *mutS* mutant host (see Table S3).

We used the same procedure to look for Beta-mediated recombination intermediates that form on replicating plasmids when the recombinase anneals the oligonucleotide to single-strand regions present at the DNA replication fork. Here, after coelectroporating pLT60 with either oligonucleotide, the amount of outgrowth was limited to only 1 h before DNA isolation, in order to preserve at least some of the recombination intermediates. Under limited plasmid replication conditions, we were also able to detect heteroduplex intermediates. For oligonucleotide LT217, which generates an unrepaired C-C mismatch when annealed to its target on the plasmid, the frequency of Kan^r^ colonies among total Amp^r^ transformants was identical when scored in either the Mut^+^ or the *mutS* mutant host. The recombinant frequency was 10-fold higher than that found in the absence of plasmid replication (Fig. 4A); some stimulation in frequency by DNA replication is expected. For oligonucleotide LT213, which forms a well-repaired G-G mismatch when paired to its target, the number of Kan^r^ recombinant colonies scored in a Mut^+^ host was reduced ~10-fold relative to that observed in a *mutS* mutant host (Fig. 4B). Hence, our data show that the DNA isolation and transformation procedure we used preserves heteroduplex plasmid intermediates that have not yet replicated.

A similar analysis was performed for the recombinase-independent *polA* mutant strain LT1715. Figure 4C shows that plasmid DNA recovered from experiments with LT1715 targeting plasmid pLT60 with either of the two oligonucleotides transforms the Mut^+^ and *mutS* mutant DH10B hosts with equal efficiency. Here, we did not detect G-G mispair heteroduplex molecules from plasmid pLT60 by oligonucleotide LT213 crosses. The data show that these recombinants, which do not depend on DNA replication, are not subject to MMR. The mechanism of their formation remains to be determined. Since LT1715 is RecA^+^, this result provides additional evidence that RecA is not responsible for heteroduplex molecules present in the plasmid DNA recovered from the Beta- and RecT-mediated experiments described above.

### Recombination of a linear dsDNA to form a circular molecule.

**FIG 5:**
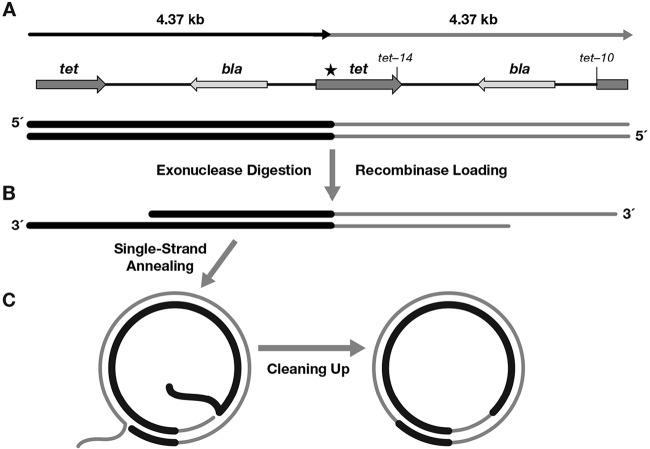
Linear dimer recombination assay. (A) The linear dimer substrate is depicted, with genes and mutations indicated. Each of the two *tet* mutations generates XhoI restriction sites. Normal PstI sites are located in the *bla* genes. The dimer is made linear by digestion at a unique BamHI site; the star indicates the location of a second defective BamHI site. (B) The 5′-to-3′ dsDNA exonuclease, either λ Exo or Rac RecE, degrades the 5′ strands of the linear dsDNA to reveal 3′ overhangs to which the λ Beta or Rac RecT recombinase binds. (C) The recombinase anneals the internal complementary ssDNA regions to form a circular monomeric plasmid intermediate with 3′ ssDNA tails. Host functions presumably remove the long single-stranded 3′ ends, with DNA ligase sealing the resulting nicks.

In the previous experiments with a circular plasmid, DNA replication provided an ssDNA target to which the Beta and RecT recombinases could anneal a single-stranded oligonucleotide. We also tested a linear dsDNA plasmid substrate on which the recombinases work in concert with their exonuclease partners to promote intramolecular recombination (Fig. 5), forming circular plasmids. We expect that this recombination will proceed by single-strand annealing, as it requires both the exonuclease activity to degrade the 5′ chains of the dsDNA substrate and the single-strand recombinase activity to anneal the resulting 3′ ssDNA complementary segments (Fig. 5). Initiation of pBR322 plasmid replication requires a supercoiled substrate (47–49), and DNA replication cannot occur until recombination-dependent circularization occurs. Hence, this linear plasmid DNA is another type of nonreplicating substrate.

### Plasmid substrate for linear DNA recombination.

The linear substrate is a directly repeated dimer of pBR322 (28) and thus has nearly 4.4 kb of tandem direct repeat homology. The linear dimer has two wild-type copies of the ampicillin resistance gene *bla* and a different mutation in each of the two copies of the tetracycline resistance gene *tet*; each of these unique mutations introduces an XhoI restriction site (Fig. 5). Symington et al. (28) showed that the RecET system efficiently recombines this type of substrate to form circular monomers and that the configuration of *tet* mutations in the final products can be determined by restriction analysis.

### Red and RecET recombine a linear dimer with high efficiency.

The Red system was expressed in *recA* mutant host HME71 or SIMD101, and the RecET system was expressed in SIMD95 or SIMD99. A dimer plasmid DNA, either linear or circular, was introduced by electroporation, and the percentage of viable cells with Amp^r^ plasmids was determined in the presence or absence of recombinase expression (Table 1). Control experiments showed that the number of transformants from the linear DNA preparation was extremely low when the phage recombination systems were not expressed (Table 1). These rare Amp^r^ transformants carried unrecombined dimer plasmids still containing the BamHI site, suggesting that they had escaped BamHI digestion and were never linearized. When either recombination system was induced at 42ºC, circular Amp^r^ plasmids, predominately monomers, were recovered with greatly enhanced frequencies. Under these recombination-proficient conditions, the linear plasmid transformation efficiency approached that of the supercoiled dimer plasmid control, indicating a high frequency of recombination (Table 1).

**TABLE 1:** Dimer plasmid DNA transformation efficiencies

Temp (ºC), condition, and DNA type	Transformation efficiency^a^	
	Red	RecET
32, uninduced		
Supercoiled	3.5	4.8
Linear	0.021	0.013
42, induced		
Supercoiled	1.6	2.9
Linear	0.70	1.04

a Transformation efficiency is expressed as the percentage of viable cells that were Amp^r^ transformants. The values obtained with the supercoiled dimer control plasmid are presented for comparison with those obtained with the linear dimer plasmid. The number of replicates in each experiment ranged between four and seven, with an average standard deviation of 49%.

### Dimer recombination products differ for Red and RecET.

Plasmid DNA from the linear dimer experiments was isolated and digested with XhoI and PstI to determine the pattern of marker inheritance (Table 2; Fig. 5) (see Materials and Methods). Analysis of the linear dimer recombination products shows that the pattern of marker inheritance differs for the two systems (Table 3). Interpreting the results as products of single-strand annealing, the Red system usually (~88% of the time) degraded the linear substrate asymmetrically, from one end or the other, and those molecules that experienced substantial degradation from both ends to generate the *tet-14* recombinant occurred at a much lower frequency (10.7%). In contrast, nearly a third (31.1%) of the plasmid molecules isolated from the RecET experiment contained the *tet-14* recombinant generated by symmetric resection, as if digestion of both DNA ends is more concerted. Butland et al. (50) identified protein-protein interactions between RecE and DNA ligase. If DNA ligase is also part of the RecET recombination complex, it may promote the completion of recombination intermediates by sealing nicks.

**TABLE 2:** Recombinant plasmids formed from linear dimer transformation

Active recombination system	No. of plasmids/total (%)				
	Monomers^a^	Symmetric resection (*tet-14*)^a^	Resection from left (*tet-10 tet-14*)	Resection from right (Tet^+^)	Other (*tet-10*)
Red	55/60 (91.7)	6/55 (10.9)	24/55 (43.6)	25/55 (45.5)	0/55
RecET	61/84 (72.6)	19/61 (31.1)	26/61 (42.6)	15/61 (24.6)	1/61 (1.6)

a The number of higher-order forms is significantly higher for RecET (*P* = 0.0051 [Fisher exact test]). The *tet-14* class is significantly higher for RecET (*P* = 0.012 [Fisher exact test]).

**TABLE 3:** *E. coli* K-12 strains used in this study

Strain	Genotype	Source or reference
DH5α	F^−^ φ80d*lacZ*ΔM15 Δ(*lacZYA-argF*)*U169 deoR recA1 endA1 hsdR17*(r_K_^−^ m_K_^+^) *phoA supE44* λ^−^ *thi-1 gyrA96 relA1*	Invitrogen
DH10B	F^−^ *mcrA* Δ(*mrr*-*hsdRMS*-*mcrBC*) φ80*lacZ*ΔM15 Δ*lacX74 recA1 endA1 araD139* Δ(*ara leu*)*7697 galU galK rpsL nupG* λ^−^ *tonA*	Invitrogen
HME6	W3110 Δ(*argF-lac*)*U169 galK_tyr145UAG_* [λ*c*I857 Δ(*cro-bioA*)]	3
HME68	W3110 Δ(*argF-lac*)*U169 galK_tyr145UAG_* *mutS*<>*cat* [λ*c*I857 Δ(*cro-bioA*)]	41
HME69	W3110 Δ(*argF-lac*)*U169 galK_tyr145UAG_* *mutS*<>*cat* [λ*c*I857 Δ(*cro-bioA*)] *polA resA1*::Tn*10*	34
HME71	HME6 Δ(*srlA-recA*)::Tn*10*	41
LT1712	SIMD89 *polA resA1*::Tn*10*	This study
LT1714	W3110 *mutS*<>*cat*	This study
LT1715	W3110 *mutS*<>*cat polA resA1*::Tn*10*	This study
LT1533	DH10B *mutS*<>*cat*	This study
SIMD89	HME6 [λ(*int-c*III) *recT*] *mutS*<>*cat*	17
SIMD95	HME6 [λ(*int-c*III) *gam recE*^a^ recT] Δ(*srlA-recA*)::Tn*10*	This study
SIMD99	HME6 [λ(*int-c*III) *gam recE*^a^ recT] *recA*<>*speC*	This study
SIMD101	HME6 *recA*<>*speC*	This study

a The *recE* gene is full length, encoding a protein of 866 amino acids.

Both the Red and RecET systems have highly processive exonucleases (14, 51) capable of degrading an entire strand of the linear dimer plasmid without dissociation. There is evidence suggesting (37, 52) that the Red system, when provided with a dsDNA molecule, degrades one of the two strands entirely, and the remaining single strand is the active recombination substrate. If an entire DNA strand of the linear dsDNA dimer substrate is degraded, circularization could no longer occur via annealing of the complementary strands, as no complementarity would exist. However, our linear dimer data demonstrate that the λ Red system does not always process dsDNAs to ssDNA intermediates; instead, our results are consistent with the more classical model (53) of partial Exo-mediated digestion of each 5′ strand of the linear dsDNA molecules, followed by Beta-mediated single-strand annealing of complementary regions within the single-stranded overhangs. This pathway is extremely efficient for either Red or RecET, with ~50% and ~30% of the linear DNA molecules becoming recombinant, respectively.

The linear dimer plasmid substrate is a nonreplicating substrate, yet both the Red and RecET systems recombine it to form circular monomers proficiently. Taken together with the results of our other experiments using an oligonucleotide to target a nonreplicating circular plasmid, these results demonstrate that the DNA replication requirement proposed for Red-mediated recombineering (33, 36, 37) depends on the DNA substrate and applies only to circular DNA molecules. Our experiments are consistent with both the Red and RecET systems acting predominately by single-strand annealing. The RecT-mediated oligonucleotide recombination frequency on freely replicating plasmids is less robust than that mediated by Beta, suggesting that RecT recombination occurs less often at single-stranded gaps present at the DNA replication fork then does Red recombination. To explain this difference, we suggest that the Beta protein may be better able to displace the single-strand binding (SSB) protein from a DNA replication fork. While we cannot rigorously rule out a low level of strand invasion in our circular-plasmid–oligonucleotide crosses, any strand invasion must be barely above the level of the background recombination occurring in the absence of recombinases.

## MATERIALS AND METHODS

### Bacterial strains, plasmids, and oligonucleotides.

The *E. coli* strains used in the experiments described here are listed in Table 3. DH5α (Invitrogen) was used as a *recA* mutant host for preparation of high-quality plasmid DNA. Oligonucleotides used for strain construction (see Text S1 in the supplemental material) and recombination studies were procured from Integrated DNA Technologies and supplied as salt free but otherwise unpurified; for their sequences, see Table S1 in the supplemental material. Agarose gel electrophoresis of plasmid DNAs was done in Tris-acetate-EDTA (TAE) buffer, routinely with 0.7 to 0.8% agarose.

### Recombineering methodology targeting circular plasmids.

Recombineering was done according to established procedures, with strains defective for methyl-directed MMR, except as noted below (54–56). For plasmid-oligonucleotide crosses, 20 ng of monomer plasmid DNA was coelectroporated with 5 pmol of oligonucleotide. This amount of plasmid gives a high but not saturating transformation efficiency (41). In order to generate a sufficient amount of plasmid DNA for analysis, five independent electroporations of identical 1-ml reaction mixtures were pooled. These pooled reaction mixtures were diluted 10-fold into L broth (LB) and grown in a 32°C shaking H_2_O bath for 3.5 h. When DNA replication was allowed, cultures were diluted and plated directly on L agar plates to determine viable cell counts, L agar plates containing ampicillin at 100 μg/ml (LB-Amp100) to determine plasmid transformation efficiency, and L agar plates containing kanamycin at 30 μg/ml to determine the recombinant frequency. When *polA* mutant hosts are used to monitor replication-independent events, direct plating of the cultures cannot be used to monitor plasmid recombination occurring in the absence of replication. In order to score recombinants from the replication-blocked crosses, after allowing time for recombination, bacterial cells were washed four times in LB to remove extracellular DNA, twice in a 30-ml volume and twice in 1 ml, followed by an additional 1-ml water wash, and then frozen at −20°C prior to DNA isolation. Plasmid DNA was isolated with a miniprep kit (Qiagen) and suspended in 30 µl of distilled H_2_O (dH_2_O). DNA (1 to 5 µl) was introduced by electrotransformation into either commercial electrocompetent DH10B Mega-X cells (Invitrogen) or LT1533 (DH10B *mutS*<>*cat*) cells, and the cells were allowed to recover for 2 h in 1 ml of LB in a 30°C roller and then diluted and plated appropriately. Recombination frequencies were normalized to the number of Kan^r^ colonies per 10^8^ Amp^r^ transformants.

On average, about 5- to 10-fold less plasmid DNA was recovered from *polA* mutant hosts than from PolA^+^ hosts; this was compensated for by introducing a larger volume of plasmid DNA from the mutant host into DH10B. In either case, about 5 ng of plasmid DNA was typically electroporated into DH10B, resulting in an Amp^r^ plasmid transformation efficiency of about 0.5% of the viable cells. Control experiments (described in Text S1 in the supplemental material) were done to determine whether the extracellular plasmid and oligonucleotide could survive the washing procedure and contribute to recombinant formation in the second host, DH10B. In no case did we recover Kan^r^ plasmids, demonstrating that the washing procedure adequately removed excess nucleic acids.

### Plasmid substrate for dsDNA linear dimer recombination.

Plasmid pRDK41 (57) was obtained from the Coli Genetic Stock Center: http://cgsc.biology.yale.edu. This pBR322-derived plasmid dimer contains two intact *bla* genes and two *tet* genes, each with a mutation, one modifying the N terminus, *tet-10*, and the other modifying the C terminus, *tet-14.* Each mutation introduces an XhoI site (28). The BamHI site near the *tet-14* allele was mutated with oligonucleotide LT807 for recombineering. This allowed the dimer plasmid to be digested at the remaining unique BamHI site to create the linear dimer recombination substrate.

### Linear plasmid recombination methodology.

The linear dimer DNA was suspended at 10 ng/µl, and 1 µl was introduced by electroporation into *recA* mutant cells expressing the phage recombination systems. Control experiments (Table 2) show that with 10 ng of supercoiled dimer plasmid DNA, 1 to 5% of the total viable cells were transformed. After a 2-h recovery period in 1 ml of LB at 30°C with aeration, cultures were diluted appropriately and plated on LB to determine the total number of viable cells and on LB-Amp100 to score plasmid transformants. Control experiments without induction of the recombination systems or with Beta and RecT expressed in the absence of their partner exonucleases confirmed that expression of both the recombinase and exonuclease was required for recombination activity on this substrate. For each experiment, 12 Amp^r^ colonies were purified and 5-ml cultures were grown in LB-Amp100. Plasmid DNA was isolated from these saturated cultures with a Qiagen miniprep kit; DNA was suspended in 50 µl of dH_2_O. Uncut plasmids were analyzed on 0.8% TAE agarose gels to determine the multimeric state of the plasmids. To determine the genetic markers present on each plasmid, ~0.5 µg of plasmid DNA was digested in a double digest with PstI and XhoI (New England Biolabs, Inc.) and the digests were analyzed by agarose gel electrophoresis. The restriction digests gave unique band patterns for each plasmid type. The starting dimer plasmid yields four bands of 3.6 kb, 2.35 kb, 2.0 kb, and 778 bp. The monomer recombinant plasmid products were one of four classes: the wild-type *tet* allele, a single band of 4.36 kb; the *tet-10* allele, two bands of 3.59 kb and 778 bp; the *tet-14* allele, two bands of 2.35 kb and 2.0 kb; and the *tet-10 tet-14* allele, three bands of 2.35 kb, 1.25 kb, and 778 bp.

## SUPPLEMENTAL MATERIAL

Text S1  Supplemental materials and methods. Bacterial strain constructions and additional control experiments are described. Download Text S1, DOCX file, 0.1 MB

Table S1  Primer sequences. The DNA sequences of the single-stranded oligonucleotides used in the experiments described here are listed. Table S1, DOCX file, 0.1 MB

Table S2  Recombination frequencies in experiments repairing a point mutation on a replicating plasmid with ssDNA oligonucleotides. Data for Red Beta, Rac RecT, and cells lacking a phage recombinase are included. Table S2, DOCX file, 0.1 MB

Table S3  Recombination frequencies in experiments repairing a point mutation on a nonreplicating plasmid with ssDNA oligonucleotides. Data for Red Beta, Rac RecT, and cells lacking a phage recombinase are included. Table S3, DOCX file, 0.1 MB

Table S4  Recombination frequencies in experiments repairing several adjacent mismatches on a replicating plasmid with ssDNA oligonucleotides. Data for Red Beta, Rac RecT, and cells lacking a phage recombinase are included. Table S4, DOCX file, 0.1 MB

Table S5  Recombination frequencies in experiments repairing several adjacent mismatches on a nonreplicating plasmid with ssDNA oligonucleotides. Data for Red Beta, Rac RecT, and cells lacking a phage recombinase are included. Table S5, DOCX file, 0.1 MB
