# Supplementary material for: Examining a DNA Replication Requirement for Bacteriophage λ Red- and Rac Prophage RecET-Promoted Recombination in Escherichia coli
Source: mBio. 2016 Sep 13;7(5):e01443-16. doi: 10.1128/mBio.01443-16 (PMC5021808; doi:10.1128/mBio.01443-16)
Supplement: Text S1 — Supplemental materials and methods. Bacterial strain constructions and additional control experiments are described. Download [file mbo004162980s1.docx]

**SUPPLEMENTAL MATERIAL**

**S1: SUPPORTING MATERIALS AND METHODS**

Construction of pLT62: Plasmid pLT62 was made from pLT61 by recombineering using oligo LT518. Following the electroporation and nonselective recovery at 30ºC for two hours, the 1ml cells were diluted to 5ml in L broth containing 100μg/ml Ampicillin and grown overnight. The culture was diluted and plated on L-plates to obtain single colonies, which were screened for sensitivity to Kanamycin by replicate plating. Two candidates were obtained from approximately 400 colonies, a frequency of 0.5%. Plasmid DNA was isolated with Qiagen kits, analyzed to identify a monomer species, and cleaved with the restriction enzyme XhoI to demonstrate the presence of a new restriction site, showing that the oligo had been incorporated. Sequencing confirmed the presence of the modification.

Detailed description of control experiments: The following questions were addressed by control experiments: 1) After DNA electroporation of the first host, does extracellular lagging strand oligo (LT217) persist through the washing regime, subsequently introduced by transformation into DH10B, and contribute to recombination there? To address this question, HME69 was induced for the Red system, made electro-competent and 20ng pLT62 plasmid was introduced by electroporation. After electroporation, 5 pmoles oligo was added to the electroporation mix, followed by 1 ml of LB. 2) Similarly, can intracellular lagging strand oligo persist and be subsequently introduced into DH10B by transformation, allowing recombinant formation in DH10B? Here HME69 was induced for Red, made electro-competent and 5 pmoles oligo was introduced by electroporation. After electroporation, 20ng pLT62 plasmid was added to the electroporation mix, followed by 1 ml of LB. 3) Do external plasmid-lagging strand oligo mixes persist through the washing steps, be introduced into DH10, and form recombinants? HME69 was induced for Red, made electro-competent the cells were treated with electroporation. After electroporation, 20ng pLT62 plasmid and 5pmoles oligo were added to the electroporation mix, followed by 1 ml of LB. In all cases, five identical independent electroporation mixes were pooled, diluted ten-fold into a total volume of 50 ml LB, and outgrown in a shaking water bath for 3.5hr at 32°C. Cells were concentrated by centrifugation and washed twice in 30 ml LB, transferred to a micro-centrifuge tube and washed two more times, once in 1 ml LB and once in 1 ml dH_2_O. Pellets were frozen until use, DNA was isolated using a Qiagen mini-prep kit, suspended in 30μl dH_2_O, and 1μl was introduced into DH10B by electroporation. In no case were KanR colonies recovered from any of these procedures.

**Bacterial strain constructions for linear dimer recombination assay:** SIMD101 was made by moving a *recA* null spectinomycin resistant allele, *recA<>spec*, into HME6 by P1 transduction. The Rac RecET expression system resident in SIMD95 and SIMD99 was made from strain SIMD49 (1), which contains the *recT* gene under *pL* control expressed from the *c*III ribosome-binding site. First, SIMD49 was transformed with pSIM18, which expresses the Red recombineering functions (2), and using recombineering, the selectable/counter-selectable *cat-sacB* cassette was inserted upstream of *recT* and subsequently replaced by the entire *recE* gene such that *recE* is expressed from the *c*III ribosome-binding site and the *recE* and *recT* genes have the same coupling as in the Rac prophage. This strain was cured of pSIM18 to create SIMD63 (3). To make LT1795, which expresses λ *gam* as well as *recET*, SIMD63 was further modified by inserting *cat-sacB* upstream of *recE* using the IPTG-inducible Red recombineering plasmid pKM208 (4), and subsequently replacing the *cat-sacB* cassette with *gam*. The recombineering plasmid was cured from this strain to yield LT1795. All insertions were confirmed to be mutation-free by DNA sequencing. Further details of strain constructions and oligo sequences are available upon request.

**S1 References**

1. **Datta S, Costantino N, Zhou X, Court DL.** 2007. Identification and analysis of recombineering functions from Gram-negative and Gram-positive bacteria and their phages. Proc Natl Acad Sci **105:**1626-1631.

2. **Datta S, Costantino N, Court DL.** 2006. A set of recombineering plasmids for gram-negative bacteria. Gene **379:**109-115.

3. **Thomason LC, Sawitzke JA, Li X, Costantino N, Court DL.** 2014. Recombineering: genetic engineering in bacteria using homologous recombination. Curr Protoc Mol Biol **106:**1.16.1-1.16.39.

4. **Murphy KC, Campellone KG.** 2003. Lambda Red-mediated recombinogenic engineering of enterohemorrhagic and enteropathogenic *E. coli.* BMC Mol. Biol*.***4:**11.
