# Supplementary material for: Examining a DNA Replication Requirement for Bacteriophage λ Red- and Rac Prophage RecET-Promoted Recombination in Escherichia coli
Source: mBio. 2016 Sep 13;7(5):e01443-16. doi: 10.1128/mBio.01443-16 (PMC5021808; doi:10.1128/mBio.01443-16)
Supplement: Table S1 — Primer sequences. The DNA sequences of the single-stranded oligonucleotides used in the experiments described here are listed. [file mbo004162980st1.docx]

**Table S1. Primer sequences**

| Primer | Sequence (5’ ->3’) | Comments |
| --- | --- | --- |
| LT217 | CGTGATAATGTCGGGCAATCAGGCGCCACAATCtaccGATTGTATGGGAAGCCCGATGCGCCAGAGTTGTT | lagging strand oligo, to repair pLT60 to Kan^+^ |
| LT213 | AACAACTCTGGCGCATCGGGCTTCCCATACAATCggtaGATTGTGGCGCCTGATTGCCCGACATTATCACG | leading strand oligo to repair pLT60 to Kan^+^ |
| LT518 | CGTGATAATGTCGGGCAATCAGGCGCCACAATCtcgagGATTGTATGGGAAGCCCGATGCGCCAGAGTTGTT | lagging strand, to make multiple mismatch mutation on pLT60 in *kan* |
| LT807 | CGACTACGCGATCATGGCGACCACACCCGTCCTGTGGATTTTGTACGCCGGACGCATCGTGGCCGGCATCA | lagging strand, to mutate *BamH*I restriction site in plasmid pRDK41 |
