# Supplementary material for: Examining a DNA Replication Requirement for Bacteriophage λ Red- and Rac Prophage RecET-Promoted Recombination in Escherichia coli
Source: mBio. 2016 Sep 13;7(5):e01443-16. doi: 10.1128/mBio.01443-16 (PMC5021808; doi:10.1128/mBio.01443-16)
Supplement: Table S2 — Recombination frequencies in experiments repairing a point mutation on a replicating plasmid with ssDNA oligonucleotides. Data for Red Beta, Rac RecT, and cells lacking a phage recombinase are included. [file mbo004162980st2.docx]

**Table S2. Repair point mutation on pLT60 with ssDNA oligos, replication allowed^1^**

| Recombination function | Lagging-strand  LT217 | | Leading-strand  LT213 | | Lag/Lead bias |
| --- | --- | --- | --- | --- | --- |
|  | Efficiency^2^ | fold reduced wrt Red | Efficiency^2^ | fold reduced wrt Red |  |
| **Beta Exo Gam** |  |  |  |  |  |
| plated directly | 3.4x10^7^ |  | 2.4x10^6^ |  | 14.2 |
| scored in DH10B | 1.2x10^7^ |  | 6.1x10^5^ |  | 19.7 |
| **RecT** |  |  |  |  |  |
| plated directly | 3.0x10^6^ | 11 | 3.2x10^5^ | 7.5 | 9.2 |
| scored in DH10B | 2.9x10^5^ | 41 | 2.9x10^4^ | 125 | 10.0 |
| **no recombinase** |  |  |  |  |  |
| plated directly | 1.8x10^3^ | 1.9x10^4^ | 1.0x10^3^ | 2.4x10^3^ | 1.8 |
| scored in DH10B | 6.7x10^2^ | 1.8x10^4^ | 4.8x10^2^ | 3.6x10^2^ | 1.4 |

^1^ All data entries are the average of three independent repeats of the experiment with an average standard error of the mean (s.e.m.) of 33%.

^2^KanR/10^8^ AmpR colonies
