# Supplementary material for: Examining a DNA Replication Requirement for Bacteriophage λ Red- and Rac Prophage RecET-Promoted Recombination in Escherichia coli
Source: mBio. 2016 Sep 13;7(5):e01443-16. doi: 10.1128/mBio.01443-16 (PMC5021808; doi:10.1128/mBio.01443-16)
Supplement: Table S3 — Recombination frequencies in experiments repairing a point mutation on a nonreplicating plasmid with ssDNA oligonucleotides. Data for Red Beta, Rac RecT, and cells lacking a phage recombinase are included. [file mbo004162980st3.docx]

**Table S3. Repair point mutation on pLT60 with ssDNA oligos, replication disallowed^1,2^**

| Recombination function | Lagging-strand  LT217 | | Leading-strand  LT213 | | Lag/Lead bias |
| --- | --- | --- | --- | --- | --- |
|  | Efficiency^3^ | replication block effect | Efficiency^3^ | replication block effect |  |
| **Beta Exo Gam** |  |  |  |  |  |
| DH10B | 1.5x10^4^ | 800↓ | 1.1x10^3^ | 555↓ | 13.6 |
| DH10B *mutS* | 1.5x10^4^ | 800↓ | 1.3x10^4^ | 47↓ | 1.2 |
| **RecT** |  |  |  |  |  |
| DH10B | 5.2x10^3^ | 56↓ | 2.7x10^3^ | 10.7↓ | 1.9 |
| DH10B *mutS* | 1.6x10^4^ | 17.5↓ | 1.6x10^4^ | 2.1↓ | 1 |
| **no recombinase** |  |  |  |  |  |
| DH10B | 1.3x10^3^ | 1.9↑ | 1.6x10^3^ | 3.3↑ | 0.8 |
| DH10B *mutS* | 4.4x10^3^ | 6.6↑ | 2.1x10^3^ | 4.4↑ | 2.1 |

^1^Plasmid DNA was isolated and introduced into the indicated host by electroporation.

^2^All data entries are the average of three independent repeats of the experiment with an average standard error of the mean (s.e.m.) of 41%.

^3^KanR/10^8^ AmpR colonies
