# Supplementary material for: Examining a DNA Replication Requirement for Bacteriophage λ Red- and Rac Prophage RecET-Promoted Recombination in Escherichia coli
Source: mBio. 2016 Sep 13;7(5):e01443-16. doi: 10.1128/mBio.01443-16 (PMC5021808; doi:10.1128/mBio.01443-16)
Supplement: Table S4 — Recombination frequencies in experiments repairing several adjacent mismatches on a replicating plasmid with ssDNA oligonucleotides. Data for Red Beta, Rac RecT, and cells lacking a phage recombinase are included. [file mbo004162980st4.docx]

**Table S4. Repair multiple mispair on pLT62 with ssDNA oligos, replication allowed^1^**

| Recombination function | Lagging-strand  LT217 | | Leading-strand  LT213 | | Lag/Lead bias |
| --- | --- | --- | --- | --- | --- |
|  | Efficiency^2^ | fold reduced wrt Red | Efficiency^2^ | fold reduced wrt Red |  |
| **Beta Exo Gam** |  |  |  |  |  |
| plated directly | 8.2x10^6^ |  | 7.8x10^5^ |  | 10.5 |
| scored in DH10B | 9.9x10^6^ |  | 5.0x10^5^ |  | 19.8 |
| **RecT** |  |  |  |  |  |
| plated directly | 1.6x10^6^ | 5 | 1.5x10^5^ | 5 | 10 |
| scored in DH10B | 2.8x10^5^ | 35 | 1.8x10^4^ | 27 | 16 |
| **no recombinase** |  |  |  |  |  |
| plated directly  standard [oligo] | 1.3x10^2^ | 6.3x10^4^ | 5.5x10^1^ | 1.4x10^4^ | 2.4 |
| scored in DH10B  standard [oligo] | 5.0x10^1^ | 1.2x10^5^ | 8.4x10^1^ | 6.0x10^3^ | 0.6 |
| plated directly  10x high [oligo] | 5.4x10^2^ | 1.5x10^4^ | 3.3x10^2^ | 1.5x10^3^ | 1.6 |
| scored in DH10B  10x high [oligo] | 1.6x10^3^ | 6.1x10^3^ | 1.0x10^3^ | 5.0x10^2^ | 1.6 |

^1^ All data entries are the average of three independent repeats of the experiment with an average standard error of the mean (s.e.m.) of 33%.

^2^KanR/10^8^ AmpR colonies
