# Supplementary material for: Examining a DNA Replication Requirement for Bacteriophage λ Red- and Rac Prophage RecET-Promoted Recombination in Escherichia coli
Source: mBio. 2016 Sep 13;7(5):e01443-16. doi: 10.1128/mBio.01443-16 (PMC5021808; doi:10.1128/mBio.01443-16)
Supplement: Table S5 — Recombination frequencies in experiments repairing several adjacent mismatches on a nonreplicating plasmid with ssDNA oligonucleotides. Data for Red Beta, Rac RecT, and cells lacking a phage recombinase are included. [file mbo004162980st5.docx]

**Table S5. Repair multiple mispair on pLT62 with ssDNA oligos, replication disallowed^1,2^**

| Recombination function | Lagging-strand  LT217 | | Leading-strand  LT213 | | Lag/Lead bias |
| --- | --- | --- | --- | --- | --- |
|  | Efficiency^3^ | fold effect of replication block | Efficiency^3^ | fold effect of replication block |  |
| **Beta Exo Gam** |  |  |  |  |  |
| DH10B  standard [oligo] | 4.8x10^3^ | 2062↓ | 4.0x10^3^ | 125↓ | 1.2 |
| DH10B  10x high [oligo] | 6.0x10^3^ | 1650↓ | 3.7x10^3^ | 135↓ | 1.6 |
| **RecT** |  |  |  |  |  |
| DH10B | 1.2x10^3^ | 233↓ | 8.6x10^2^ | 21↓ | 1.4 |
| **RecET** |  |  |  |  |  |
| DH10B | 1.0x10^4^ | 2.8↓ | 1.5x10^4^ | 3.0↑ |  |
| **no recombinase** |  |  |  |  |  |
| DH10B  standard [oligo] | 2.2x10^2^ | 4.4↑ | 4.8x10^1^ | 1.1↓ | 4.6 |
| DH10B  standard [oligo]  10x high carrier | 1.5x10^2^ | 3.0↑ | 4.2x10^1^ | 1.8↓ | 3.6 |
| DH10B  10x high [oligo] | 1.7x10^3^ | 1.1↑ | 1.0x10^3^ | NA | 1.7 |

^1^Plasmid DNA was isolated and introduced into DH10B by electroporation.

^2^All data entries are the average of at least three independent repeats of the experiment with an average standard error of the mean (s.e.m.) of 41%.

^3^KanR/10^8^ AmpR colonies
